# Unifying models of dialect spread and extinction using surface tension dynamics

**DOI:** 10.1098/rsos.171446

**Published:** 2018-01-03

**Authors:** James Burridge

**Affiliations:** Department of Mathematics, University of Portsmouth, Portsmouth, UK

**Keywords:** dialects, social systems, coarsening

## Abstract

We provide a unified mathematical explanation of two classical forms of spatial linguistic spread. The *wave* model describes the radiation of linguistic change outwards from a central focus. Changes can also jump between population centres in a process known as *hierarchical diffusion*. It has recently been proposed that the spatial evolution of dialects can be understood using surface tension at linguistic boundaries. Here we show that the inclusion of long-range interactions in the surface tension model generates both wave-like spread, and hierarchical diffusion, and that it is surface tension that is the dominant effect in deciding the stable distribution of dialect patterns. We generalize the model to allow population mixing which can induce shrinkage of linguistic domains, or destroy dialect regions from within.

## 1. Introduction

Processes which involve copying in spatial regions, such as language evolution [1], the design of Stone Age personal ornaments [2] and natural selection [3–5], produce different results in different places. It is typical for subregions to emerge in which one distinctive form of language, style of ornament or member of a taxonomic genus is the most commonly observed. These domains are typically large in comparison to the movement ranges of the interacting agents who do the copying, and are reminiscent of the domains which emerge in the physical process of *coarsening* which takes place in crystalline structures such as ferromagnets [6] and binary alloys [7]. Here, boundaries between regions with different atomic orderings feel a form of *surface tension* which causes them to smooth out over time. If we view spatial ordering at the atomic level as analogous to the emergence of distinctive local cultural or genetic structures, then it is natural to ask if our understanding of physical coarsening can be adapted to explain the spatial distribution of copying phenomena more generally.

To investigate this idea, we require observational data on the spatial distribution of the results of a copying process. An ideal example is language dialects. For centuries humans have been drawing maps of the spatial domains in which they live, creating records of their languages and sometimes combining the two to create linguistic atlases. The linguistic analogy of a physical domain with a particular atomic ordering is a region in which one linguistic form is in common use. The connection between physical and social ordering has been exploited by physicists studying social systems in many contexts [8]. In a recent paper [9], the author set out the theoretical foundations of a general theory of dialect patterns, based on a linguistic version of the ‘Ginzburg–Landau’ equation [6,10] for physical ordering. The classical Ginzburg–Landau equation predicts that domains with distinct atomic orderings will form and that the boundaries between these domains will tend to become less curved over time producing fewer, but larger domains. This process is known as *coarsening*. The linguistic version of this equation involves speaker memories, accounts for variations in population density and predicts a modified form of coarsening where boundaries between atomic orderings are now boundaries between regions where different linguistic forms are used. These boundaries are known as *isoglosses* by linguists. Solutions to this equation suggest that the spatial distribution of language may have a significant element of predictability, owing to two opposing forces which drive isoglosses. The first, surface tension, renders isoglosses smoother and straighter over time. The second, wherein isoglosses are driven down population density gradients, creates curvature. The first effect also means that stable isoglosses must emerge perpendicular to the boundaries of language areas (such as coastline, mountain ranges or country borders). Combined together, within any given language area, these influences drive many different initial patterns of language use towards one of a much smaller number of stable equilibrium configurations. An extensive discussion of the relationship between physical and human geography, and the form of these typical equilibrium states, is given in [9].

Early models of linguistic change, which neglected spatial effects, treated the history of language as a branching evolutionary tree [11]. Spatial aspects of linguistic spread were then recognized in the ‘wave model’ [12,13] in which linguistic forms spread out from a central focus (the source of a new linguistic innovation). Bloomfield [13] recognized the fronts of these waves as isoglosses which form the intersecting network observed by linguists. It is important to realize that isoglosses rarely define sharp linguistic boundaries; rather there is a transition region [1] through which a mixture of linguistic forms are observed. In the surface tension (linguistic Ginzburg–Landau (LGL)) model [9], which has its roots in Bloomfield’s concept of ‘communication density’ [13], spatial evolution is driven by local interactions between individual speakers, who have a tendency to conform to the locally most popular patterns of language use. In this model, transition regions form spontaneously, allowing isoglosses to be defined as lines which follow along the middle of these transition regions. The surface tension felt by these isoglosses and the influence of variations in population density and system shape cause them to travel like the waves. The power of the approach is that it leads to predictions about where these waves will travel, and therefore the most likely spatial pattern of language use. It is of course the case that there may be factors which inherently bias the motion isoglosses, such as the ease with which one linguistic variable may be adopted compared to another [14]. However, the direction in which such factors act at each isogloss is not known *a priori*, in the sense that if we pick an isogloss at random, we cannot know in advance the bias direction. As a consequence, the deterministic component of isogloss evolution is mainly driven by system (e.g. country) shape and population distribution: it is *geometric* in origin.

Moving beyond the wave model, linguists recognized that linguistic changes often jumped between population centres in a cascade-like process known as *hierarchical diffusion*. A famous and influential attempt to capture this effect is the ‘gravity model’ [1,15]. Here linguistic innovations can spread between population centres at a rate which depends on the square of the distance between them, and on their population sizes. This important model has been partially successful in predicting observed sequences of linguistic change [1,16–18], and offers some qualitative insight into the most likely positions of isoglosses [15]. However, the model suffers somewhat from the fact that it breaks down at the level of individual speakers, and offers no testable mathematical theory of isogloss formation or dynamics. One purpose of our work is to show that the surface tension model, with the addition of long-range interactions between speakers, is able to reproduce both the wave-like motion of isoglosses and hierarchical diffusion between population centres. The fundamental object in our theory remains the isogloss, but now, long-range interactions allow new bubbles (isogloss loops) to spontaneously form in population centres. However, once these bubbles have formed, they evolve by the surface tension effect. As a result, we suggest that although the evolution of language use exhibits both wave-like spread and hierarchical diffusion, both potentially influenced by inherent bias in isogloss movement, it is likely to have been geometrical effects associated with surface tension which matter most for the long-term historical linguistic evolution of stable populations.

Having studied the behaviour of isoglosses in some detail, we finally investigate the conditions under which they remain stable. As societies develop, their populations become increasingly mobile, with individuals and families resettling in widely separated locations multiple times during their lives. Mathematically, population mixing adds a stochastic element to the spatial distribution of language use, but may also be seen as a source of long-range interactions: when people move, they transport their linguistic memories and speech patterns with them creating linguistic links between distant locations. If people move throughout the system, and not just between major population centres, then this can induce bias towards forms of speech which are dominant in the system as a whole. To study these effects, we define a stochastic version of our model, finding that dialect regions can be abruptly destroyed when migration or mixing reaches a critical level. We also find that mixing-induced bias (as opposed to inherent bias towards one linguistic variant) in isogloss motion can, under some circumstances, lead to dialect destruction by domain shrinkage.

### 1.1. Statistical physics of language

We, here, give a brief survey of work on the statistical physics of language; the subfield to which the present paper belongs. We also set our work in a wider context.

The achievements for which Ludwig Boltzmann (1844–1906) is most famous are his methods for deriving the macroscopic equations which describe matter, beginning from minimal assumptions about the behaviour of its constituent particles. For Boltzmann these assumptions were necessarily minimal, because at the time he was making them, there was considerable doubt about the existence of atoms [19]. His approach, called ‘Statistical Physics’, showed that, in large systems, knowledge of only the most essential properties of the interacting units was often enough to understand macroscopic behaviour in a precise way. Because human societies may be viewed as large collections of interacting agents, and given Boltzmann’s success in the physical realm, it has been natural for Statistical Physicists to investigate whether large-scale aspects of collective social behaviour also follow predictable laws derivable from simple assumptions.

Our paper is on the spatial distribution of language, where the formation of distinctive local forms may be seen as a social ‘ordering’ process. There are two classic models which are the antecedents to many of the models which attempt to describe social ordering and consensus: these are the voter and Ising models [6,8]. The voter model, lacking surface tension, may be seen as the simplest lattice model of neutral evolution [20], where opinions or other characteristics are inherited with a probability which is in proportion to their current frequency. The Ising model, describing the alignment of magnetic spins within a lattice, when evolved according to Glauber dynamics [6] has a nonlinear (non-neutral) alignment rule which favours the local majority. This nonlinearity, if sufficiently strong, induces surface tension at boundaries between groups of oppositely aligned spins, causing these boundaries to straighten over time [6,7] creating an ordered state. The motion of boundaries, known as ‘coarsening’, is experimentally realized, for example, in binary alloys [7]. A number of models exist which attempt to explicitly model language evolution as a process of assigning sounds to objects [8]. For example, in the minimal form [21] of the naming game [22], speakers possess an inventory of word–object associations which they modify through simple interaction rules, eventually reaching a consensus. Like the Ising model, explicit models of language formation (including the naming game and also bitstring models [23]), and models of social ordering [8], when they are evolved on a two-dimensional grid, form a patchwork of different consensus domains. The boundaries of these domains (domain walls) feel surface tension. The ubiquity of this effect is reflected by the fact that the large-scale (coarse-grained) dynamics of these walls is the same in every case, and described by the Ginzburg–Landau equation. Ising, Landau and Ginzburg were born in 1900, 1908 and 1916, respectively. It is interesting to note that linguists had been observing similar boundaries throughout the twentieth century [13] as isoglosses, but the patterns they observed were complex and ‘tangled’: there were many different overlapping consensus regions. Recognizing the connection between isoglosses in linguistics and domain walls in condensed matter physics, and that surface tension dynamics is a macroscopic phenomenon largely independent of the details of the underlying language (or other) model, the author defined a minimal model for isogloss motion [9] based on a single assumption: that speakers tend to conform to the language they hear in the places where they spend most time. The model showed how population distribution modified surface tension, and that system boundary shape can have a powerful effect on how language in the *interior* of the system evolves: the boundaries of many different consensus regions are driven towards a set of equilibrium forms. The current paper builds on this work.

Beyond the study of the spatial distribution of language and models of individual speakers, modern word-frequency databases, derived from millions of books, have revealed new scaling relationships [24–28]. Whereas Zipf’s original law of words [29] states that the frequency of a word is an inverse power (close to one) of its frequency rank, in fact the distribution is much closer to a double (broken) power law [24,26,27], which may be explained [26] using a generalization of the Yule process, originally used to model phylogenetic trees [30]. Word-frequency databases have also revealed that fluctuations in the ranks of the most popular words have reduced over time [28], suggesting that word popularity may be determined by preferential attachment [28]. It has also been suggested that as languages expand to include more words, they also ‘cool’ in the sense that fluctuations in the frequencies with which individual words are used decay over time [27]. Techniques developed to understand the physics of quenched (frozen) disorder have also been used to explain how a focus on topics within individual texts controls fluctuations in word-frequency statistics in large groups of texts [31].

## 2. The model

We begin by deriving a ‘LGL equation’ [6] which describes how the use of language evolves in space. The spatial structure of human linguistic interactions is inherently complex, involving the daily motion of speakers and an ever-changing social network. Rather than arrive at our equation via general plausibility arguments (as in [9]), we derive it from some transparent microscopic assumptions. Although plausibility arguments have the advantage of being easier to defend because of their ambiguity, the microscopic approach exposes modelling assumptions more clearly, following the traditions of statistical physics. However, it is important to realize that, as in physics, several different microscopic models may yield the same macroscopic result. In this sense, the macroscopic equations are more fundamental.

We take as our starting point a very large collection of speakers represented as points in a two-dimensional domain. Each point is the *home* position of a speaker, and the collection of home positions may be used to form a coarse-grained density *ρ*(**r**). As she goes about her daily life, each speaker has the potential to interact with any other in the domain and forms a constantly evolving network of social contacts. Suppose we select a speaker at random from the population. If her home lies at **r**_*i*_, then the probability that she will converse with a speaker at **r**_*j*_ during time interval *δt* is written *p*_*ij*_*δt*+*o*(*δt*). We call *p*_*ij*_ the *interaction rate* between **r**_*i*_ and **r**_*j*_.

While some people have ways of life which mean that they most often interact with individuals from their local area, others may regularly interact with people from much further afield. These long-range interactions are facilitated by proximity to transport hubs, economic or cultural centres. To capture these different types of interactions we allow speakers to exhibit two types of behaviour: *metropolitan*, or not. In our model, the set of speakers who are behaving as *metropolitans* at any given time are distinguished by a high probability of communicating with each other over long distances. That is, the interaction rate between pairs of metropolitans is a much more slowly declining function of their geographical separation than the interaction rate between other pairs of speakers. We use the term *metropolitan* because speakers who have access to these long-range links tend to be concentrated in cities rather than rural locations. Although we do not model the precise mechanisms which induce these links, the phenomenon of hierarchical diffusion strongly suggests that such mechanisms do exist. To capture the links mathematically, we let **1**_*i*_ be the indicator function that the speaker at **r**_*i*_ is currently behaving as a metropolitan (**1**_*i*_=1 in this case, otherwise **1**_*i*_=0), and introduce two alternative interaction rates: a long-range form, *p*^†^, which acts between metropolitans, and a Gaussian (short-range) form, p*, which acts between all other possible pairings, so that

2.1 $$p_{ij}=(1-{\text{1}}_{i}{\text{1}}_{j})p_{ij}^{*}+{\text{1}}_{i}{\text{1}}_{j}p_{ij}^{†}.$$

**Figure 1.**
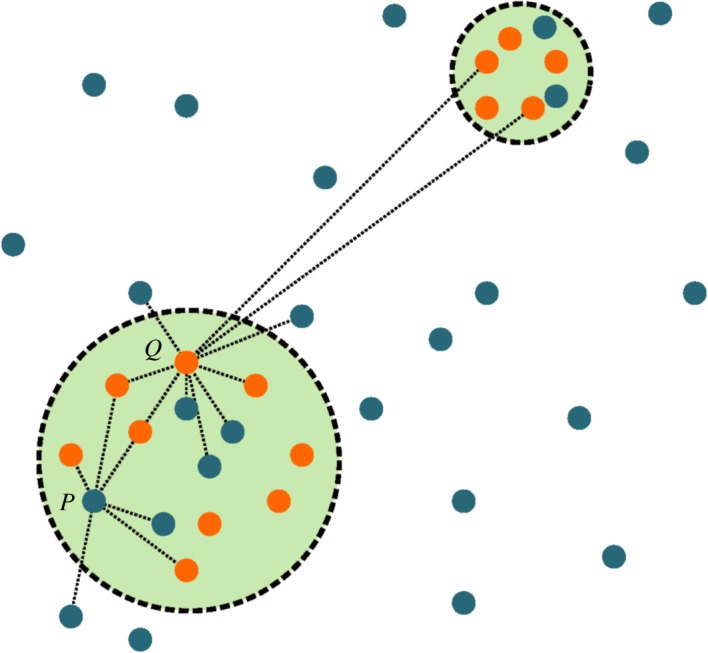
A set of possible connections in the network of linguistic interactions for two speakers *P* and *Q*. Orange dots represent speakers who are currently behaving as metropolitans and blue dots as those behaving as non-metropolitans. Green circles represent cities. Note that long-range connections can only be made between pairs of metropolitans.

Figure 1 shows an example of the set of interactions made by two different speakers over a period of time. We assume that nearby speakers exhibit similar time-averaged levels of metropolitan behaviour.

To derive macroscopic equations for the evolution of our system, we let *δS*(**r**) denote the set of speakers in a small region centred on **r** with area *δA*. We assume that |*δS*(**r**)|≈*ρ*(**r**)*δA*≫1, and define the coarse-grained fraction of the local population who are behaving as metropolitans at any given time:

2.2 $$M(\text{r}):=\frac{1}{|\delta S(\text{r})|}\sum_{i\in \delta S(\text{r})}{\text{1}}_{i}.$$

Changing speaker behaviour will cause this field to fluctuate in time, but we assume that the condition |*δS*|≫1 ensures that these fluctuations can be ignored. We also define *d*(**r**_1_,**r**_2_)≡*d*_12_ to be the ‘travel distance’ between two points; typically, this will be the Euclidean distance, but in some cases natural barriers or varying quality of transportation may change this. Consider the group of speakers in *δS*(**r**_1_). A widely used functional form for long-range social interactions is the inverse square law (justified below). To make connection with this classical approach, we define our long-range interaction rate to be Lorentzian (Cauchy) [32]. The expected fraction of the linguistic interactions that speakers *δS*(**r**_1_) have with speakers from *δS*(**r**_2_) is then

2.3 $$\begin{array}{r} \frac{1}{N({\text{r}}_{1})|\delta S({\text{r}}_{1})|}\sum_{i\in \delta S({\text{r}}_{1})}\sum_{j\in \delta S({\text{r}}_{2})}[(1-{\text{1}}_{i}{\text{1}}_{j})p_{ij}^{*}+{\text{1}}_{i}{\text{1}}_{j}p_{ij}^{†}]=\left[\frac{1-M_{12}}{\exp[d_{12}^{2}/(2\sigma^{2})]}+\frac{M_{12}}{1+d_{12}^{2}/\gamma^{2}}\right]\frac{\rho ({\text{r}}_{2})\delta A}{N({\text{r}}_{1})}:=\psi ({\text{r}}_{1},{\text{r}}_{2})\delta A, \end{array}$$

where *M*_12_:=*M*(**r**_1_)*M*(**r**_2_). We call *ψ*(**r**_1_,**r**_2_) the *interaction density*. The constant $N({\text{r}}_{1})$ is chosen so that $\int_{R^{2}}\psi ({\text{r}}_{1},{\text{r}}_{2})\;d{\text{r}}_{2}=1$. The parameters *σ* and *γ* control, respectively, the length scales of local and metropolitan interactions.

We justify the Lorentzian interaction term in (2.3) on the following basis. Mobile phone records [33] indicate that the communication intensity between pairs of cities follows a ‘gravity law’, being proportional to the product of their populations divided by the square of their separation. The same law has been shown to hold for traffic flows between Korean cities [34]. Data on human mobility [35,36] also indicate that the distribution of human travel distances has an algebraic tail. Consistent with these observations, the long-range component of (2.3), integrated over all pairs of points in two metropolitan centres with small radii in comparison to their separation, *r*, is approximately proportional to *P*_*i*_*P*_*j*_/*r*^2^, where *P*_*i*_ and *P*_*j*_ are their populations. Gravity laws, first introduced in 1946 [37], are not without limitations [38,39], but have the virtue of mathematical simplicity and are widely used to predict population movement [39].

We represent different patterns of language use with a set of discrete linguistic variables [1]. These might be grammar rules, pronunciation choices or words. For simplicity we consider the case where each variable has two alternatives or *variants* which we label A and B. Our analysis is readily generalized to any number of alternatives, as in [9]. Consider one particular variable and let *f*(**r**,*t*) be the average frequency with which variant A is used by speakers within *δS*(**r**) at time *t*. As there are two variants, the frequency with which B is used is 1−*f*(**r**,*t*). Based on the observation that linguistic changes take place over years and decades [40] and that speakers use thousands of words per day [41], we assume that they are in possession of an accurate and up-to-date sample of current language use, weighted according to the interaction density. The sample mean of the frequency of a feature is then equal to the spatial average

2.4 $$\bar{f}(\text{r},t):=\int_{R^{2}}f({\text{r}}^{\prime },t)\psi (\text{r};{\text{r}}^{\prime })\;d{\text{r}}^{\prime }.$$

According to this definition, all speakers within *δS*(**r**) have the same spatial weighting of their samples, consistent with our earlier assumption that nearby speakers are similarly metropolitan.

A person’s geographical origins can often be guessed from the way they talk. This reflects the fact that their current use of language is a function of the linguistic interactions they have experienced in the past. If a speaker has remained in approximately the same geographical location throughout her life, then her accumulated linguistic experience may be represented as an integral of $\bar{f}(\text{r},t)$ over history, weighted to reflect the relative importance of linguistic information collected at different times in the past. Greatest mathematical tractability is achieved if the weighting is exponential, and there is some experimental evidence for this choice [42]. We refer to the historical average as a speaker’s *memory*, the coarse-grained version of which is

2.5 $$m(\text{r},t):=\int_{-\infty }^{t}\frac{e^{(s-t)/\tau }}{\tau }\bar{f}(\text{r},s)\;ds,$$

where *τ* measures the timescale over which memories retain their importance. Finally, we define how current language use is based on this memory. There is considerable evidence that human beings have a tendency to conform to the behaviour exhibited by the majority of their social group [43–45]. Indeed, such conforming behaviour is a necessary condition for the formation of locally distinctive culture. A simple way to capture such conformity is to suppose that the current frequency with which a feature is used is a sigmoidal function, *p*(*m*), of a speaker’s memory for its frequency in the past

2.6 $$p(m):=\frac{m^{\alpha \beta }}{m^{\alpha \beta }+{\left(1-m^{\alpha }\right)}^{\beta }}=f,$$

where *β*≥1 (the ‘conformity’ number) measures the extent to which speakers conform to local language use and *α* models any inherent bias towards the feature in question. When *α*>1, speakers need to have heard variant A more than half the time in the past in order to use it with equal frequency to B in the present. The case *α*=*β*=1 corresponds to no conformity or inherent bias. Differentiating (2.5) with respect to time, then rescaling time to units of length *τ*, we have

2.7 $$\dot{m}(\text{r},t)=\bar{f}(\text{r},t)-m(\text{r},t).$$

This is our time-dependent LGL equation. As in [9], we define an isogloss to be the set of points for which $f(\text{r},t)=\frac{1}{2}$, so that when such a line is crossed the most commonly used local linguistic variant changes. For reference, table 1 lists the definitions of paramaters and symbols used throughout the paper.

**Table 1.** Collected definitions of parameters and symbols appearing in the paper.

parameter or symbol	definition
*f*(**r**,*t*)	frequency of variant A
$\bar{f}(\text{r},t)$	spatial average frequency of variant A
*m*(**r**,*t*)	memory for variant A
*ρ*(**r**)	population density
*σ*	local interaction range (short)
*γ*	range of interactions between metropolitans (long)
*β*	tendency to *conform* to local majority
*α*	inherent bias towards one variant (apart from bias towards the majority)
*κ*	isogloss curvature
*v*	isogloss velocity
*ω*	peak city population density
*R*	city radius
*B*	radius of city hinterland
*ϵ*	mixing rate within linguistic domain
*ν*	immigration rate into linguistic domain

## 3. Wave-like spread and hierarchical diffusion

To explore the behaviour of solutions to our LGL equation (2.7), we consider a collection of cities with Gaussian population densities. The density for the *i*th city is defined to be

**Figure 2.**
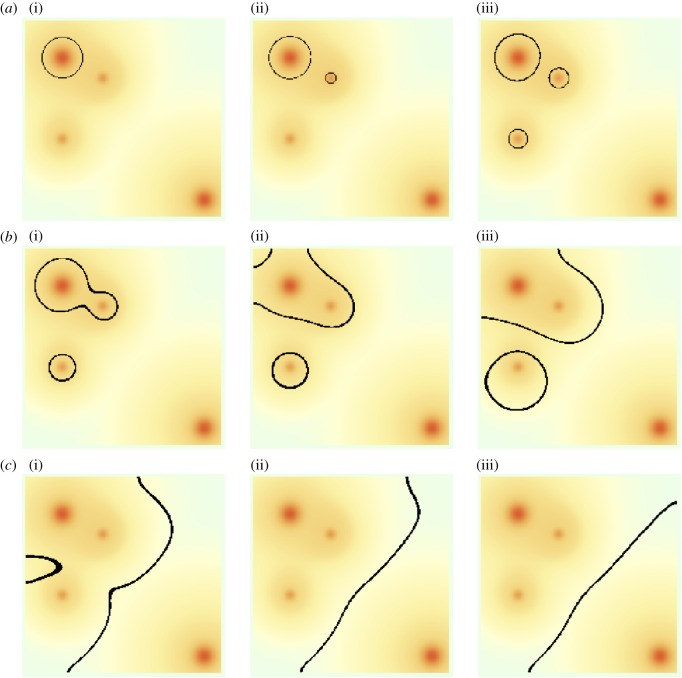
Evolution of a 200×200 system containing four cities. Background colour indicates population density with deep orange/red representing higher-density areas and lighter shades indicating lower density. Large cities have *ω*_*i*_=*R*_*i*_=10, while small ones have *ω*_*j*_=*R*_*j*_=5. In all cases, the background radius is five times the city radius. The system is initialized with one large city (a(i)) using variant A and all others using B. Model parameters are $\sigma =2\sqrt{2},\gamma =25,\beta =1.1$. There is no inherent bias for or against either variant (*α*=1). Black lines represent isoglosses: $f(x,y,t)=\frac{1}{2}$. Images correspond to evolution times *t*=1,3,10 (*a*(i–iii)); then *t*=40,80,120 *and* 160,200,300, respectively.

3.1 $$\rho_{i}(\text{r}):=\omega_{i}\exp\left\{\frac{|\text{r}-{\text{r}}_{i}{|}^{2}}{R_{i}^{2}}\right\}+\exp\left\{\frac{|\text{r}-{\text{r}}_{i}{|}^{2}}{B_{i}^{2}}\right\},$$

where *ω*_*i*_>1 and 0<*R*_*i*_<*B*_*i*_. The first term represents the city itself, while the second term is a more slowly decaying background population which dominates outside the main metropolitan area, representing smaller outlying towns and villages. We refer to *R*_*i*_ as the ‘radius’ of the city. In figure 2, we follow the progress of variant A, which is initially in common use within one of the large cities. In this example, we assume that all speakers within a distance *R*_*i*_ of each city centre behave entirely as metropolitans and can therefore make long-range connections to metropolitans in the centres of other cities, as well as to non-metropolitans sufficiently close to edges of their own cities. Long-range connections mean that a speaker within a city which is sufficiently small and close to the large city will experience a greater number of interactions with the large city than with their own. From figure 2 we see that, as a result, variant A jumps to the satellites of the large city. This is an example of *hierarchical diffusion*. Once these initial jumps have taken place, the isogloss loops surrounding the new variant in each city begin to spread out. This spreading effect may be understood using a linguistic analogue of the Allen–Cahn equation [7] (describing coarsening in binary alloys), giving the velocity of an isogloss in terms of its curvature and the local population density

3.2 $$v=-\sigma^{2}\beta \left\{\frac{\kappa }{2}+\frac{\nabla \rho \cdot \hat{\text{g}}}{\rho }\right\},$$

where $\hat{\text{g}}$ is a unit vector normal to the isogloss and *κ* is its curvature (see [9] for a derivation of this result). The curvature term in (3.2) acts to shorten isoglosses in the same way that surface tension acts to reduce the area of bubbles. The population gradient term causes isoglosses to migrate away from regions of high population density. In figure 2, we see that these effects act in combination to create isogloss waves which eventually encircle the three cities to which variant A had spread. An equilibrium is finally reached due to the repulsion effect of the other large city. This example serves to illustrate how linguistic forms can travel by hierarchical diffusion (jumps induced by long-range interaction) and as waves, through short-range interaction. If the density of metropolitans is greatest in cities, then the final spatial distribution of a linguistic form will typically be determined by short-range effects (surface tension, creating coarsening).

We now provide an dynamical analogue of Trudgill’s gravity model [1,15] for the hierarchical diffusion of a linguistic variable. Suppose we have *N* cities, each with a metropolitan core with radius *cR*_*i*_ in which all speakers are metropolitan. Let *P*_*i*_ denote the population of this core. If the length scales of short- and long-range interactions obey: *σ*<*cR*_*i*_<*γ*, then the fraction of a centrally located speaker’s interactions that are with other speakers from his own city is

3.3 $$\begin{array}{r} \frac{2\pi }{N({\text{r}}_{i})}\left[\int_{0}^{cR_{i}}\frac{r\rho_{i}(r)}{1+r^{2}/\gamma^{2}}\;dr+\int_{cR_{i}}^{\infty }r\;e^{-r^{2}/2\sigma^{2}}\rho_{i}(r)\;dr\right]=\frac{1}{N({\text{r}}_{i})}\left[P_{i}+O\left(\frac{{\left(cR_{i}\right)}^{2}}{\gamma^{2}}\right)+O\left(\frac{e^{-{\left(cR_{i}\right)}^{2}/2\sigma^{2}}}{2\sigma^{2}+R_{i}^{2}}\right)\right], \end{array}$$

where *ρ*_*i*_(*r*) is the population density at a distance *r* from the centre. If *σ*≪*cR*_*i*_≪*γ*, then the dominant term in (3.3) is $P_{i}/N({\text{r}}_{i})$. Let us assume that language use within each core is approximately uniform and given by *f*_*i*_(*t*), where *i* indexes the city, and that the distances *r*_*ij*_ between cities are large compared to their radii. A speaker at the centre of city *i* will have the following language sample:

3.4 $${\bar{f}}_{i}(t)\approx \frac{1}{N_{i}}\left(P_{i}f_{i}(t)+\sum_{j\neq i}\frac{P_{j}f_{j}(t)}{1+r_{ij}^{2}/\gamma^{2}}\right),$$

where $N_{i}=Pi+\sum_{j}P_{j}/(1+r_{ij}^{2}/\gamma^{2})$. The memories of metropolitan speakers, therefore, obey a set of coupled ordinary differential equations (ODEs)

3.5 $${\dot{m}}_{i}(t)={\bar{f}}_{i}(t)-m_{i}(t),\;i\in \{1,2,\ldots ,N\}.$$

**Figure 3.**
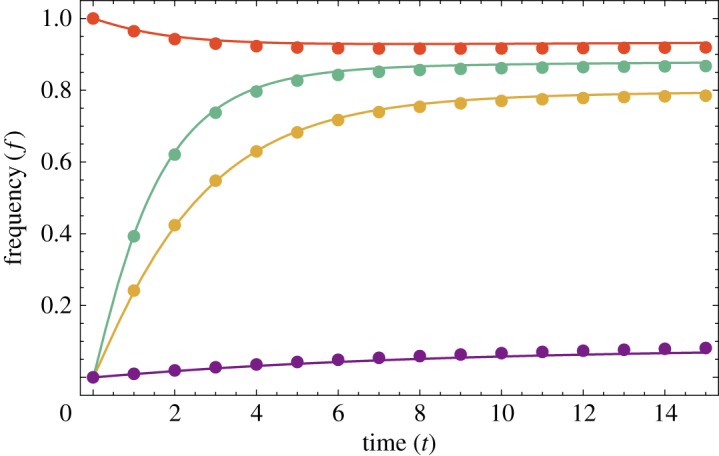
Comparison of frequencies *f*_*i*_(*t*) of variant A at the centres of the four cities in figure 2 (plotted as points) to the solution of the set of approximate ODEs (3.5) (plotted as lines). Colour scheme: red, large city which begins with variant A; green, first city to adopt A in figure 2; yellow, second city to adopt A and purple, large city which never fully adopts A.

These equations describe the hierarchical diffusion of a linguistic feature from one city to another: the variant *jumps* from one city centre to the next without travelling through the rural areas in between. It is important to realize, however, that, in this simplified form of the model, *only* the dynamics of cities are considered explicitly and not wave-like spread through rural areas. From figure 3, we see that their solutions closely approximate the behaviour of the full model for the given parameter values. We note that the equations become exact as σ→0 and γ→∞.

**Figure 4.**
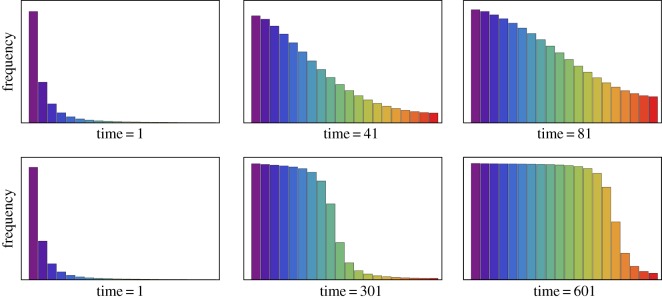
Snapshots of solutions to ODEs (3.5) for a line of 20 cities, each separated from the next by 50 distance units. Each bar represents frequency of variant A within a single city. City populations are *P*_1_=30 and *P*_*i*>1_=5, with initial conditions *m*_1_(0)=1, *m*_*i*>1_(0)=0. In both cases, the length scale of long-range interactions is *γ*=25. For the upper sequence, *β*=1.1, and for lower sequence *β*=1.5.

The approximate equations (3.5) may be used to provide some insight into the importance of conformity in hierarchical diffusion. Consider a line of cities with equal spacing between each. Let the first city on the line be significantly larger than the others and consider the progress of linguistic variant A which is initially used only in this first city. Provided this city is sufficiently large, the variant will jump to nearby smaller cities and then onwards. We illustrate the dynamics of this process in figure 4 for two different values of conformity. When conformity is low, outside influence in each city (from other cities) produces a greater shift in local language use before there is a full switch to a new modal variant. This allows an early but partial conversion to the new variant in distant cities, before the full conversion takes place. For high conformity, the full switch between variants is more dramatic: there is only a small preliminary shift in language use. In the high-conformity limit (β→∞) the switch is discontinuous: there is no change in language use at all, until the influence of the opposing variant hits a critical level, at which point there is a full switch to the new variant. From figure 4, we see that high conformity produces a diffusion effect closer to the intuitive idea of a series of jumps where the new variant spreads to one new city at a time. We emphasize that, in figure 4, although the bars representing usage frequencies in each city are adjacent, the cities themselves are separated by significant distances, and the linguistic variant has jumped from one city to the next in the sense that it has not passed through the rural areas in between.

## 4. The effects of inherent bias

We now consider the effect of an inherent bias towards one variant, on an isogloss passing through a non-metropolitan area. This bias is controlled by the parameter *α* introduced in equation (2.6), and represents factors which, *in the absence of a locally dominant variant*, would nudge speakers towards using one variant in favour of another. We call this *inherent* bias to distinguish it from social conformity, controlled by *β*, which acts to push speakers towards the locally most common forms of speech. In this sense, conformity may be viewed as a different form of bias, towards the majority. Factors which would be viewed as inducing inherent bias in our model are, for example, a desire to sound like a certain social subgroup which is not necessarily in the majority, or the tendency to opt for a simpler pronunciation when two alternatives are available.

When an isogloss lies in a non-metropolitan area, assuming that population density changes approximately linearly on the scale of short-range interactions (|∇^2^*ρ*|≪*σ*^2^), our LGL equation (2.7) reduces to the form studied in [9]

4.1 $$\dot{m}=f-m+\frac{\sigma^{2}}{2}\frac{\nabla^{2}(\rho f)}{\rho }.$$

Here we evaluated the integral (2.4) over local interactions using the saddle point method [46] (a standard method used to approximate integrals). To isolate the effects of bias from those of population density variations and curvature, we consider a straight isogloss passing through a region of constant population density. Under these conditions, in the absence of inherent bias towards one or other variant, its velocity would be zero. We use standard Cartesian coordinates to describe position so that **r**=(*x*,*y*) and, without loss of generality, we suppose that the line of the isogloss is described by a constant *x* coordinate (it runs vertically), so the memory field depends only on *x* and not *y*. The motion of the isogloss will then be described by a travelling wave of the form

4.2 m(x,t)=ϕ(x−vt),

where the function *ϕ* describes the shape of the wavefront (the transition region). Without loss of generality, we assume that the isogloss is located at *x*=0 when *t*=0, so $\phi (0)=\frac{1}{2}$. Substitution of this travelling wave solution into (4.1) yields, after a change of variable *u*=*x*−*vt*,

4.3 $$-v\phi^{\prime }(u)=p[\phi (u)]-\phi (u)+\frac{\sigma^{2}}{2}\{p^{\prime }[\phi (u)]\phi^{\prime \prime }(u)+p^{\prime \prime }[\phi (u)](\phi^{\prime }{\left(u)\right)}^{2}\}.$$

We may obtain a linear approximation for the velocity *v* in terms of the bias *α* by noting that, as α→1, the shape of the travelling wavefront must converge to the shape of a stationary transition region. In this limit, the gradient of the frequency field at the position of the isogloss obeys [9]

4.4 $$|\;f^{\prime }|\to \sqrt{\frac{4\ln2-1}{6}}\frac{\sqrt{\beta -1}}{\sigma }\;\text{as}\;\beta \to 1,$$

so *ϕ*′(0)=| *f*′|/*β* (see equation (2.6)). We now replace *ϕ* in equation (4.3) with its form in the limit α→1, set *u*=0 and solve for *v*. Both memory and frequency fields have an inflection point at the isogloss in this limit, so *ϕ*′′(0)=0 and

4.5 $$|v|\approx \frac{p(1/2)-1/2}{|\;f^{\prime }|/\beta }+\frac{\sigma^{2}}{2}\frac{|\;f^{\prime }|}{\beta }p^{\prime \prime }\left(\frac{1}{2}\right).$$

Expanding this expression to linear order about *α*=1, we obtain

4.6 $$|v|\approx \left[\frac{0.6376\beta^{2}\sigma }{\sqrt{\beta -1}}+O\left(\sqrt{\beta -1}\right)\right](\alpha -1).$$

For small bias, curvature and population gradients, the transition region will be close to its equilibrium shape for a straight isogloss, so we expect the contributions to the total velocity from these three effects to be additive, giving

4.7 $$v\approx -\sigma^{2}\beta \left\{\frac{\kappa }{2}+\frac{\nabla \rho \cdot \hat{\text{g}}}{\rho }\right\}-\frac{0.6376\beta^{2}\sigma (\alpha -1)}{\sqrt{\beta -1}}.$$

We can test expression (4.7) by computing the stable radius of an isogloss surrounding a city with population density

4.8 $$\rho (r)=\omega \exp\left\{-\frac{r^{2}}{R^{2}}\right\}.$$

Taking $R=30\sqrt{2},$ we find that when *α*=1.01,*β*=1.1 and *σ*=5, the stable radius of the isogloss, predicted by finding the radius for which *v*=0 in equation (4.7), is 63.7, compared with 64.0 estimated by simulation. The stable radius without bias is 75.2. Intuitively, the bias against the city variable has caused its domain to shrink, but in doing so the isogloss has moved to a region of greater population gradient, increasing the outward expansive force to the point where it balances the bias. The significance of this observation is that although bias can cause individual linguistic regions to shrink or expand, the motion may be arrested by other effects. The stable geographical configuration may therefore be determined by population gradients and system shape as well as inherent bias towards certain variants. In fact, were bias the only determiner of isogloss motion, then we would expect to see very little linguistic diversity because linguistic variables towards which there was an inherent bias would expand until they filled the entire system.

**Figure 5.**
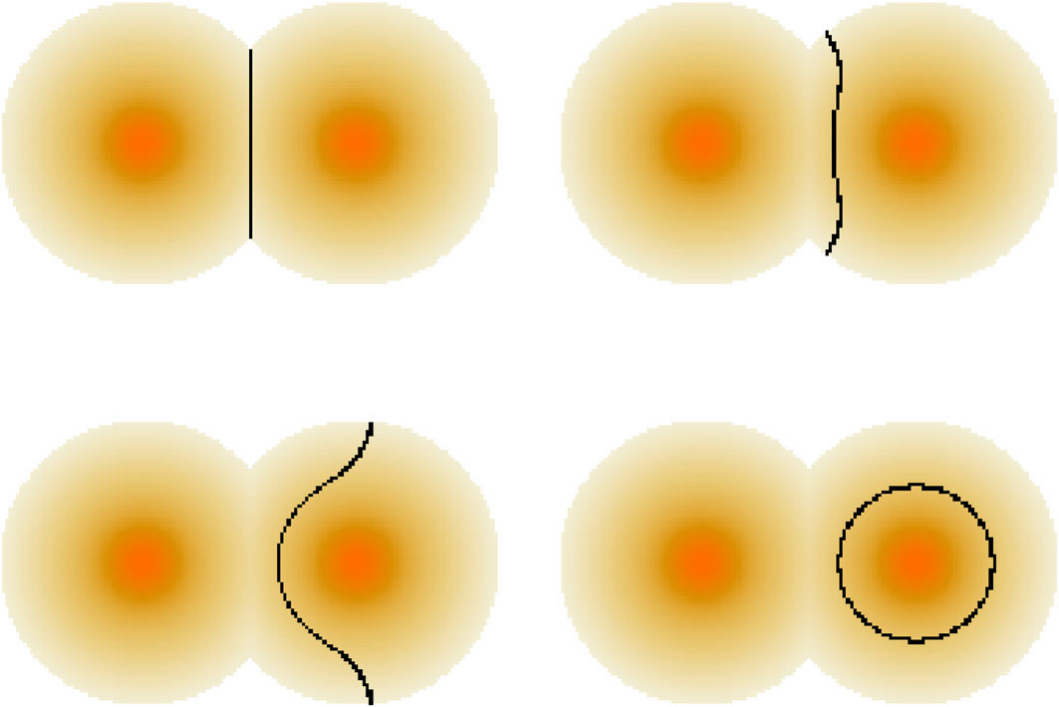
Stable isogloss positions for different bias values in a lobed system (lobe diameter 80 units) containing two cities with Gaussian population densities $\rho (r)=\omega \exp(-r^{2}/R^{2})$ with *ω*=10,*R*=10. Interaction range *σ*=4, conformity *β*=1.1 and bias values *α*∈{1,1.035,1.04,1.05}.

To illustrate how system shape and population distribution interact with inherent bias, we have simulated a linguistic domain consisting of two lobes, each containing a city (figure 5). In the absence of bias, an isogloss connecting the two boundary indentations will be stable. Stability arises because any perturbation away from this position would force the isogloss either to meet the system boundary at an incident angle other than *π*/2 (90^°^), or to become curved. As surface tension (associated with coarsening) acts to reduce curvature, and to re-establish normal incident angles between isoglosses and system boundaries, it follows that the perturbed isogloss would migrate back to its stable state. It is argued in [9] that this mechanism is responsible for the large number of isoglosses observed to connect the Wash and the Severn estuary (two major coastal indentations) in Great Britain. If we now introduce bias in favour of the variant used in the left-hand lobe, then from figure 5 we see that the isogloss is pushed away from its zero-bias equilibrium. This perturbation creates isogloss curvature which, along with the population gradient of the right-hand city, opposes the bias, creating a new equilibrium. Increasing bias further pushes the isogloss deeper into the right-hand lobe, and at a critical level of bias the isogloss detaches from the system boundary and surrounds the city. The essential point to note here is that small biases perturb stable isogloss positions, but the effects of surface tension and population gradients remain visible.

The origin of bias towards certain linguistic features may be social in origin. For example, the pronunciation of a particular vowel may indicate one’s social class [1]. If membership of this class confers prestige, this may induce a social pressure towards this pronunciation, even if the class that uses it does not represent the majority of local speakers. However, different speakers may desire, consciously or otherwise, affiliation with different social groups, so bias direction may depend on the group to which one belongs [47]. In principle, our model could be generalized to allow different social classes to exist in the same location, but we do not pursue this here. Apart from social factors, inherent bias (as defined in our model) may also arise due to the presence of other linguistic variables. For example, the southward spread of the northeast Polish form, śfyńa, of the word, świnia, for hog may be explained [14] by the fact that the phonological system of southern speakers allowed them easily to produce the northern form, but northerners had no such option. It is important, also, to distinguish between conformity, which induces bias towards the most common local forms of speech—a form of community solidarity—and the bias defined by our parameter *α*. This latter form only has a tangible effect when there are already two variants in common use (i.e. in the transition region near an isogloss); otherwise, it would be overwhelmed by conformity.

## 5. Application to Italian dialects

Previously, we applied our surface tension model to the English dialects of Great Britain [9]. To further compare our model to real linguistic data, we now consider the case of a second country—Italy. The elongated shape of this country, its mountainous regions, producing large variations in population density and complex coastline, give a large number of distinct equilibrium solutions to our evolution equation. We consider the extent to which these solutions are able to match the distribution of the usually recognized subdivisions of Italian dialects [48].

Repulsion of isoglosses from cities means that stable isoglosses tend to lie in regions where most interactions are local. We also see from figure 2 that the timescale over which long-range effects dominate isogloss evolution is short in comparison to the times over which surface tension and population gradients take hold. For this reason, we assume that stable patterns of language use should largely be determined by short-range interactions, and we work with the purely local version of the model, defined by equation (4.1). This approach may be further justified by noting that long-range connectivity is likely to have been less significant in the past than it is today. We lack detailed knowledge of how the spatial distribution of language use evolved in the centuries before dialect maps were created. We, therefore, average over a wide range of randomized initial spatial distributions of language use. Predictability arises because surface tension, population distribution and the shape of the language area draw many different early language distributions towards a much smaller number of later stage configurations.

Population density is approximated using the GEOSTAT 2011 dataset [49], a standardized 1 km^2^ resolution Europe-wide population grid generated using census data from participating countries. These data are only an approximation to the population distribution during the historical period when dialects were forming. However, because most population centres have been in existence for centuries at least, and because the model depends only on population gradients and not on absolute population sizes, modern population distribution provides a reasonable proxy for the past. Before use in the model, the raw population data are smoothed by replacing the population density spike at each census location with a normally distributed density with standard deviation *σ*_s_ (skewed to account for boundary conditions such as coastline). This smoothing distance, *σ*_s_, is a free parameter of the model but we argue that it should be comparable, but somewhat smaller, than the interaction range. To see this, suppose that linguistic interactions were infrequent and only ever involved one speaker leaving her census location and arriving at that of another for a conversation. In this case, the population density that each speaker would perceive would be the raw census data. In reality, speakers may often meet when they are both away from home, so they perceive a smoothed out population distribution.

**Figure 6.**
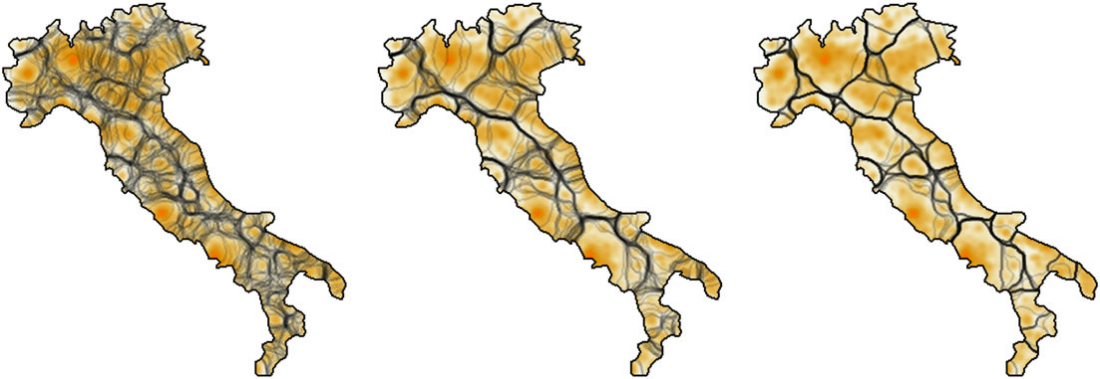
Superposition of Italian isoglosses generated by 50 solutions of equation (4.1), each with different initial conditions. Model parameters are *σ*= 12 km, *σ*_s_=6 km and grid spacing is 3 km. Solutions shown at *t*=10,50,100 (time measured in units of memory length *τ*). Each isogloss is shown as a semi-transparent line, so overlapping isoglosses produce darker lines.

Following [9], we predict dialect areas by first generating a large sample of stable isoglosses. These are obtained by numerically solving equation (4.1) on a discrete grid of points representing the language areas of interest, using a different randomized initial condition for each solution. Figure 6 shows a superposition of isoglosses for Italy, generated by this method, at different stages of evolution. We note the similarity between the pattern of isoglosses, and the regions (regioni) defined in the Constitution of the Italian Republic, 1948 [50]. The randomization procedure consists of setting the memory value in each grid square equal to a random number uniformly generated from the interval [0,1]. We do not make any claims as to the realism of these initial conditions but note instead that they allow us to statistically explore a wide range of possible historical states. The fact that many different early isogloss configurations converge towards certain highly probable curves indicates that the precise details of early history are not important, at least within our model. Having generated a set of isoglosses, a large number (≈10^4^) of geographical locations are selected and the set of modal variants, one for each possible initial condition, are recorded. This set of variants determines the local ‘dialect’. Ward’s hierarchical clustering algorithm [51] (available in the R language [52]) is then used to group together geographical locations which have similar dialects. We compare to our dialect maps by cutting the synthetic hierarchical tree into *n*_*c*_ clusters, where *n*_*c*_ is the number of real dialects.

**Figure 7.**
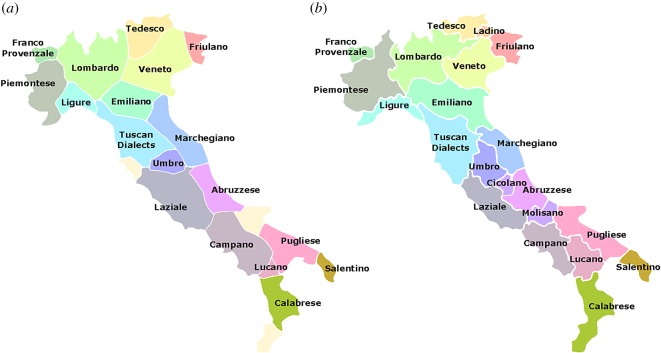
(*a*) Main dialect areas identified by Ward cluster analysis (*n*_*c*_=21) of 50 synthetic equilibrium isoglosses with *σ*=12 km, *σ*_s_=6 km, *t*=200, starting from randomized initial conditions. Grid spacing of 3 km. (*b*) Main Italian dialect areas identified from Pellegrini’s map and listed in [48], p. 234. Population weighted overlap between two maps: *WOL*=79%; non-weighted overlap: *OL*=66%. Synthetic regions with no clear counterpart in Pellegrini’s map are coloured beige and have been left unlabelled.

Italian is a *Romance language* [48] meaning that it is descended from the Vulgar (common) Latin spoken during the time of the Roman Empire. Latin originated in the central western region of the Italian Peninsula, Latium, in which Rome was founded. As the Romans colonized the peninsula, their language and culture were influenced by contact with other peoples (including Etruscans, Ligurians, Illyrians, Pheonicians and Greeks) [50], creating regionally distinct forms of speech. The collapse of the Roman Empire and consequent loss of centralized political control allowed differences between these local dialects to become further exaggerated. These differences persisted well into the twentieth century; in 1945, 50% of Italians spoke only a dialect [50]. Various settlements and occupations of the peninsula also introduced elements of Spanish, French and Austrian [53]. Although it is possible to classify the dialects of Italy into hundreds of different local variants, there are 18 main Italo-Romance subdivisions [48]. These are shown on the map in figure 7 which has been adapted from the dialect map of Giovan Pellegrini [54] (1977). Also shown are the German dialect region (Tedesco), the Franco-Provencale and Ladin regions giving a total of *n*_*c*_=21 dialect areas within the Italian peninsula. Pellegrini uses a two-level alpha-numeric classification system, with codes for each sub-dialect marked on the map. The first level of classification corresponds to the main subdivisions marked in figure 7 (except for Tuscan, which is further subdivided by Pellegrini). These areas are in close but not exact correspondence with Italian administrative regions (regioni). For example, the Emilia-Romagna region is classified by Pellegrini as using the Emiliano dialect, whereas the Apulia region is divided into Pugliese and Salentino. As boundary lines are not given on the map, we interpret regional borders as dialect boundaries except where a region contains more than one of the 21 main dialect subdivisions. In this case, we add approximate border lines based on Pellegrini’s labelling. Pellegrini’s further subdivisions draw subtler distinctions between regional varieties, producing a more detailed patchwork of language use. The precise geographical details of this pattern are not accessible because no boundary lines between them are provided. Indeed, where these regions are comparable in size to the width of isogloss transition regions, isoglosses are a less meaningful representation of spatial variation, which may be better described by a continuum of variation [55]. Although our focus is on the main dialect divisions, we note that such finer scale variations can be generated by reducing the interaction range, *σ*, which narrows the width of transition regions.

**Figure 8.**
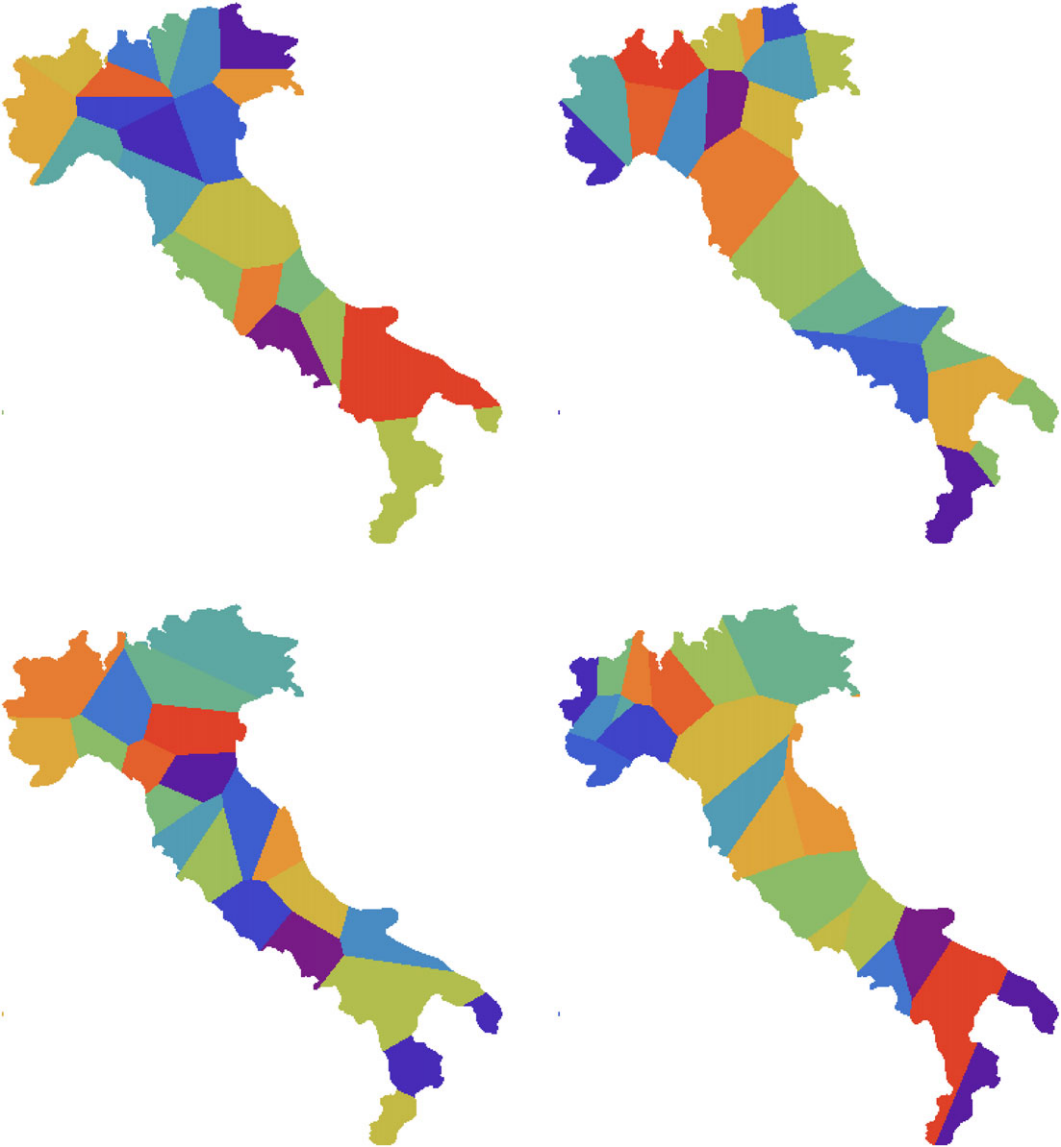
Four Voronoi tessellations of Italy into *n*_*c*_=21 cells, using points selected uniformly at random from within the land area. The mean overlap between Voronoi tesselations and Pellegrini’s map is 52±4%.

Also shown in figure 7 are the results of our synthetic cluster analysis using a set of 50 synthetic isoglosses, examples of which can be seen in figure 6. To compare the similarity of these maps, we have computed the ‘overlap’ (*OL*=66%) as the fraction of land area which is classified as belonging to the same dialect in both maps. The ‘weighted overlap’ (*WOL*=79%) accounts for population density, giving the fraction of individuals who will be classified as living in the same dialect area on both maps. We provide a measure of the significance of these figures by defining a null model, in which dialect areas are generated as a Voronoi tessellation with *n*_*c*_ cells (figure 8). This model is ‘null’ because it is not based on any assumptions about the process by which dialects are formed. By labelling the cells of this tessellation so as to maximize overlap with the real map (using the Hungarian algorithm [56]), we find for a set of five realizations of the null model that OL ≈52±4%. Our synthetic dialect areas are therefore significantly closer to Pellegrini’s map than the null model, by approximately four times the standard error of the null model in the case of raw overlap (OL).

## 6. Mixing and dialect death

Stable dialects take considerable time to form, and rely on speakers being geographically rooted. As the world becomes richer, more educated and better connected, people have become increasingly mobile throughout their lives, and may resettle multiple times [57,58]. When this happens, they carry linguistic information from one place to another, effectively mixing up the geographical distribution of linguistic variables [59]. We now examine the effect that this process will have on the stability of dialects in our model. We also consider the effect of all speakers, not just city dwellers, having long-range interactions.

Some of the effects of population resettlement are driven by the stochasticity inherent in the process of migration. To capture these effects, we move away from our coarse-grained view of the model, and simulate the behaviour of individual speakers. We arrange the home locations of our speakers on a two-dimensional lattice where each site (*x*,*y*) has probability *ρ*_*xy*_ of being the home location of a speaker. We assume periodic boundary conditions and model interactions between speakers as an embedded network [39,60] where each pair of sites is connected with probability

6.1 $$H(\Delta x,\Delta y)=\exp\left\{-\frac{\Delta x^{2}+\Delta y^{2}}{2\sigma^{2}}\right\},$$

where (*Δx*,*Δy*) is the displacement of one from the other.

The dynamics of individual speakers are defined in an analogous way to the coarse-grained dynamics, with the embedded network replacing the interaction density. We write *m*_*xy*_(*t*) for the memory of the speaker located at site (*x*,*y*) and let 〈*x*,*y*〉 denote the set of sites to which (*x*,*y*) is connected, then

6.2 $${\dot{m}}_{xy}(t)=\frac{\sum_{\left(i,j)\in \langle x,y\right\rangle }f_{ij}(t)}{|\langle x,y\rangle |}-m_{xy}(t)=:h_{xy}[\{m_{ij}(t)\}].$$

This evolution equation holds while the population of speakers are settled. For later notational convenience we have defined the right-hand side of (6.2) as a function, *h*_*xy*_, of the set of all memory values. To investigate the effects of population mixing, we consider the evolution of language use in two adjacent cities, initially using different variants. The cities, located at (*x*_1_,*y*_1_) and (*x*_2_,*y*_2_), are represented by site occupation probability

6.3 $$\rho_{xy}=\min\{(1-\rho_{0})[e^{-(\Delta x_{1}^{2}+\Delta y_{1}^{2})/R_{1}}+e^{-(\Delta x_{2}^{2}+\Delta y_{2}^{2})/R_{2}}]+\rho_{0},1\},$$

where *ρ*_0_∈[0,1] is a background population density, *R*_1_ and *R*_2_ are city radii and *Δx*_*i*_:=(*x*−*x*_*i*_), *Δy*_*i*_:=*y*−*y*_*i*_. This system serves as a microcosm for larger regions, allowing us to illustrate two distinct mechanisms by which mixing can destroy dialects. Population mixing is captured through random exchanges of the memories at two occupied sites, giving the following stochastic modification to the deterministic equation (6.2)

6.4 $$m_{xy}(t+\delta t)=\left\{ \begin{array}{ll} m_{xy}(t)+h_{xy}[\{m_{ij}(t)\}]\delta t & \text{w.p.}\;1-\epsilon \delta t,\\ m_{UV}(t) & \text{w.p.}\;\epsilon \delta t, \end{array}\right.$$

where ‘w.p.’ stands for ‘with probability’. Here the coordinates (*U*,*V*), selected by some random rule from within the set of occupied sites, are those of an exchange partner. In this paper, our random rule is to select a partner uniformly at random from the set of occupied sites. This dynamics can lead to the disappearance of variants in two ways.

### 6.1. Internal destruction

#### 6.1.1. Spontaneous internal destruction

**Figure 9.**
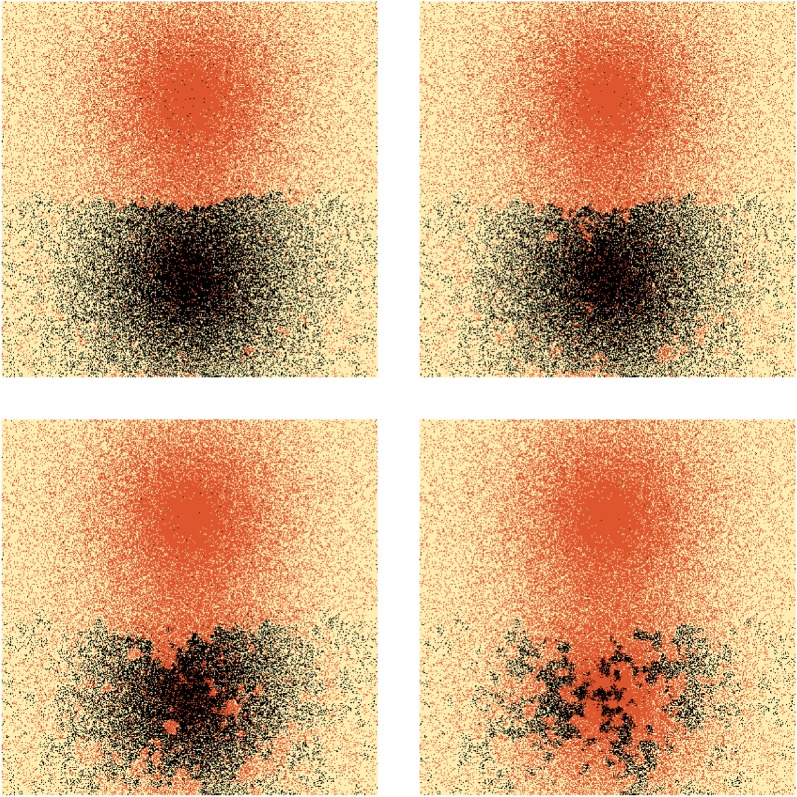
System of size 400×400 with two equal-sized cities, radius *R*=100, located at (200,100) and (200,300). Background population density is *ρ*_0_=0.1, interaction range *σ*=3 and conformity *β*=1.1. Red sites are occupied by a speaker using variant A by preference, black sites are the reverse. System shown when the mixing rate is *ϵ*∈{0.118,0.119,0.12,0.121}.

We consider a square system which is initially divided into two equal-sized stripes using different variants, creating a straight isogloss as shown in figure 9. In the centre of each stripe lies a city of radius *R*=100. In the absence of population mixing this is a stable configuration of language use. We allow the mixing rate to increase slowly over time as *ϵ*(*t*)=5×10^−4^*t* and, as a result, small fluctuations in the number of speakers using each variant appear. If one variant, say A, is used by more than half the speakers in the system, then this creates an effective bias towards A, because more A speakers are migrating into B regions than vice versa. This is *not* an inherent bias because neither variant is innately more attractive than the other. Rather, the mixing acts as a weak form of very long-range interaction (γ→∞) so that speakers are effectively globally connected through the arrival of others from all over the system. If conformity is sufficiently low, then these migrations can seed small colonies of A speakers within B regions, and the bias effect is further strengthened. This process can be seen in figure 9, where we see that the B region dissolves into a set of disconnected subregions which are eventually overcome by variant A. We describe this process as *internal destruction* because the B region was destroyed from within, rather than shrinking.

#### 6.1.2. Internal destruction by inward migration

Internal destruction may be understood in a simpler way by considering an isolated language area where initially only variant A is used, into which pure B speakers migrate, replacing existing speakers at rate *ν*, producing the following dynamics:

6.5 $$m_{xy}(t+\delta t)=\left\{ \begin{array}{ll} m_{xy}(t)+h[\{m_{ij}(t)\}]\delta t & \text{w.p.}\;1-\nu \delta t\\ 0 & \text{w.p.}\;\nu \delta t. \end{array}\right.$$

As the region is colonized, a complicated spatial pattern of speaker types will emerge, similar to that seen in figure 9. To gain a qualitative understanding of the destruction process, we make the mean field approximation

6.6 $$m_{xy}(t)\approx \frac{1}{n}\sum_{\mathrm{speakers}}m_{xy}(t):=m(t),$$

where *n* is the number of speakers in the region. From equation (6.5), we find that the mean field memory value satisfies

6.7 $$\dot{m}=\frac{m^{\beta }}{m^{\beta }+{\left(1-m\right)}^{\beta }}-(1+\nu )m.$$

**Figure 10.**
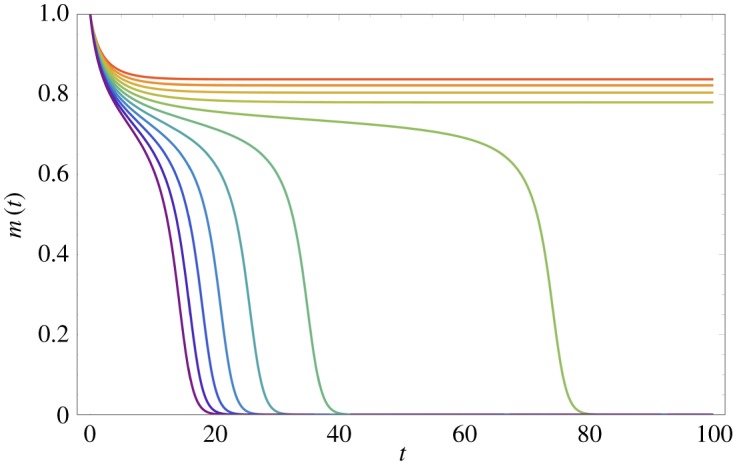
Mean field memory values obtained by solving equation (6.7) subject to initial condition *m*(0)=1, with *β*=1.5. Colours correspond to different immigration rates *ν*∈0.1,0.105,0.11,…,0.15 with red solution corresponding to *ν*=0.1 and violet to *ν*=0.15. Intermediate spectrum colours (orange, yellow, green, etc.) correspond to intermediate values of *ν*. Critical immigration rate is *ν*_*c*_=0.118 to 3 significant figures (determined by finding the value of *ν* for which the right-hand side of equation (6.7) had a repeated root in the interval *m*∈(0,1)).

For sufficiently small *ν* this equation possesses two stable fixed points (values of *m* for which $\dot{m}=0$): one at *m*=0 and another at $m^{*}>\frac{1}{2}$. Solving subject to the initial condition *m*(0)=1, we find that $m\to m^{*}$ as t→∞. Upon increasing *ν*, we find there is a critical value *ν*_*c*_ at which the non-zero fixed point disappears. In this case, the linguistic variant which was initially used by all speakers is eventually wiped out. This effect is illustrated in figure 10, which shows a family of solutions to the mean field equation for a series of values of immigration rate. By numerically determining the fixed points, we find that larger values of *β* increase the critical value *ν*_*c*_. Intuitively, where there is a greater level of conformity to a local dialect, a higher rate of immigration can be supported without that dialect being lost.

### 6.2. Destruction by isogloss migration

**Figure 11.**
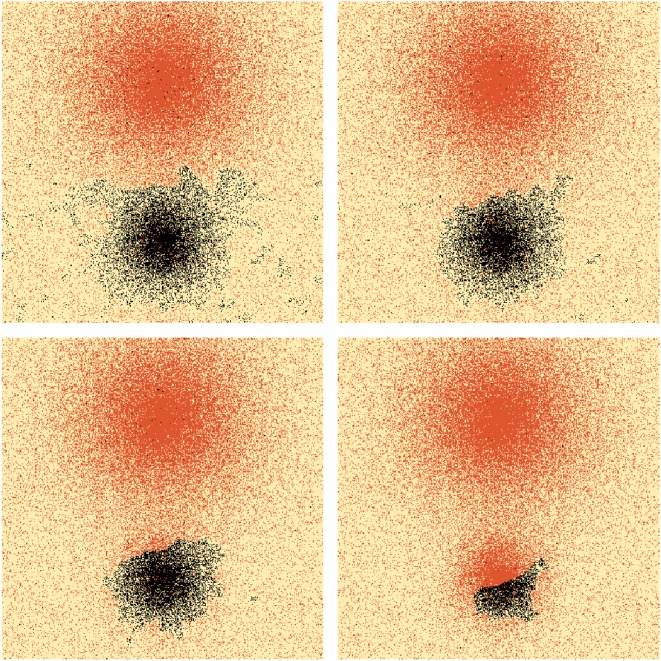
System of size 400×400 with two cities, radii *R*=100 and *R*=50, located at (200,100) and (200,300). Background population density is *ρ*_0_=0.1, interaction range *σ*=3, conformity *β*=2 and mixing rate *ϵ*=0.1. Red sites are occupied by a speaker using variant A by preference; black sites are the reverse. System initialized in stripe state, and shown at *t*=20,30,40,50.

The degree of conformity to local variants can influence the spatial mechanism by which linguistic forms disappear. To illustrate this, we consider two cities of different radii, initially using different variants separated by a straight isogloss. We raise the conformity parameter to *β*=2 and fix the mixing rate at *ϵ*=0.1, which is insufficient to destroy the small-city dialect internally. Instead, the isogloss surrounding the city shrinks as shown in figure 11, so that the city dialect remains locally distinctive until it is eventually swallowed up by the big-city variant. The shrinking process may be explained by noting that in the transition region at the edge of the shrinking dialect, both variants are used in approximately equal proportion. The influx of speakers using the big-city variant tips the balance in this region, allowing conformity to take hold and the incoming variant to become established, further shrinking the region in which the small-city variant is used. The shrinking effect may be seen as a non-inherent bias induced by mixing, which effectively creates very long-range weak interactions throughout the system. We discuss this point further in §6.3.

The question of which process: *internal destruction* or *isogloss movement* is the main driving force of spatial change, could be addressed in two ways. It is often the case that language survey data from a given geographical region is only available at one point in time. In this case, because the rate at which people adapt their speech to new forms declines as they age, a linguistic change can be detected by observing different speech patterns in the young and old. Such changes are said to have been observed in *apparent time* [40]. Internal destruction would then lead to apparent time changes throughout a dialect area, whereas isogloss movement would lead to changes only near the edge of a dialect region. It is possible that both processes take place simultaneously, in which case the relative magnitude of boundary versus internal changes would indicate the relative importance of the two effects. If two studies of a large geographical area are made at different times, then if dialect loss is taking place, we may observe both shifts in isogloss position and loss of uniformity in speech patterns within the dialect region which is disappearing. Recently developed linguistic survey methods using a mobile phone application [61], together with traditional studies such as the Survey of English dialects [62] will make such comparisons possible.

### 6.3. Universal long-range (metropolitan) interactions

We now return to our deterministic model (2.7). Here, the practical mechanism by which metropolitan interactions were induced was defined only in quite general terms. As we have noted, such interactions might be produced by migration of speakers carrying their linguistic memories with them, provided that migratory journeys collectively conformed to an inverse square distance law. Technically, this would conflict with our microscopic assumption that the locations of speakers were fixed for all time. However, if we treat the coarse-grained macroscopic evolution equation (2.7) as fundamental, and interpret the memory field *m*(**r**,*t*) as the average memory of speakers *currently* located in the neighbourhood of **r**, then migration may be seen as a legitimate source of long-range interactions in the deterministic model. If such migrations take place only between cities, then the wave and cascade effect illustrated in figure 2 will be preserved. However, if speakers outside of cities are just as mobile or socially connected as city dwellers (as was the case in our stochastic simulations), so that long-range interactions of all origins are felt throughout the system, then the situation changes.

**Figure 12.**
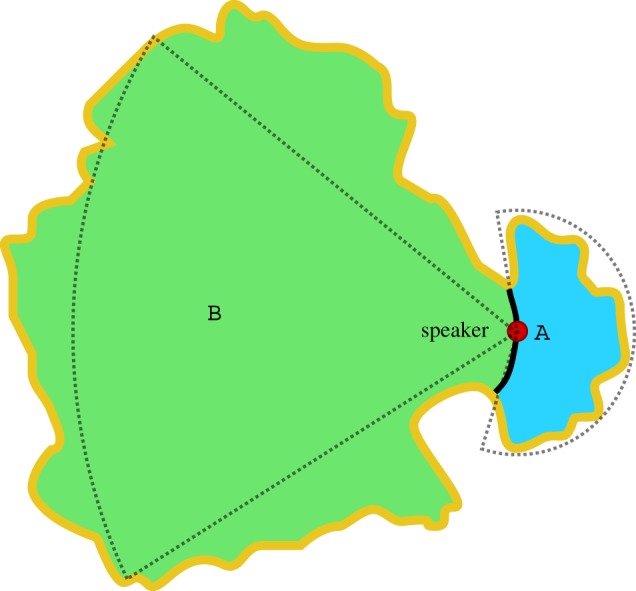
An island nation forming a language area where two variants are in use. Sectors of circles may be used to bound the domains of variant A (blue) and variant B (green) from above and below. Yellow line indicates the island’s coast.

In a system where all speakers are metropolitan, the Lorentzian term in the interaction density (2.3) induces communication between every possible pair of speakers, and is therefore computationally infeasible to simulate. Instead, we give a simple analytical argument which illustrates how universal long-range interactions change the behaviour of the system. In figure 12, we illustrate an island language area where two variants are in use. We suppose that the domain of variant A would be stable under short-range interactions and that population density is approximately constant. Consider a speaker on the isogloss separating the domains. Now suppose that all interactions are long range. We can find an upper bound for the non-normalized interaction density integrated over domain A by integrating over the smallest circle sector, radius *r*, central angle *θ*_*A*_, which bounds the domain

6.8 $$\int_{A}\frac{1}{1+{\text{r}}^{2}/\gamma^{2}}\;d\text{r}\leq \int_{0}^{r}\frac{s\theta_{A}}{1+s^{2}/\gamma^{2}}\;ds=\frac{\theta_{A}\gamma^{2}}{2}\ln\left[1+\frac{r^{2}}{\gamma^{2}}\right].$$

An equivalent calculation for domain B, but using the largest sector (defined by radius *R* and angle *θ*_*B*_) which lies inside the domain, yields the following sufficient condition for domain A to shrink:

6.9 $$\theta_{A}\ln\left(1+\frac{r^{2}}{\gamma^{2}}\right)<\theta_{B}\ln\left(1+\frac{R^{2}}{\gamma^{2}}\right).$$

From this we see that when speakers experience long-range interactions, if the B domain is big enough, then the A domain will disappear, even if the isogloss would have been stable under local interactions. In the limit γ→∞, stability is decided simply by considering the relative areas of the two domains. This example serves to illustrate that although long-range interactions do not preclude the existence of isoglosses, they effectively induce a form of non-inherent bias towards the dominant variant in the system as a whole. Their stability is therefore determined by the global properties of the system.

## 7. Conclusion

We have set out a simple geometrical picture of the way in which the spatial distribution of language use evolves, developed from the surface tension model described in [9]. The central object of study has been the isogloss, analogous to the domain walls of condensed matter physics. By identifying two length scales of interaction, we have shown that both wave-like spread and hierarchical diffusion observed by linguists may be understood in a unified way. The model shows that jumps of linguistic forms between cities are followed by slower, surface tension-driven evolution.

The effect of inherent bias towards one linguistic variant has been incorporated into a single ‘Allen–Cahn’ equation for isogloss velocity. This form of bias may be seen as one of three effects which determine final stable isogloss shapes, the others being surface tension and population gradients. The existence of stable isoglosses implies that any inherent bias must be comparable to the strength of these other effects (or even weaker and potentially non-existent), and its presence will perturb the stable isogloss curves predicted on the basis of population density and system shape alone. The isoglosses which are generated by the model have been shown to have predictive power in determining the distribution of real dialects.

Finally, we have shown how population mixing and long-range interactions can destroy local dialects either by overwhelming local linguistic variants when immigration is above a critical level, or by creating a non-inherent bias in the direction of isogloss motion arising from the fact that speakers are weakly connected to the system as a whole. We have also seen that dialect destruction by shrinkage may be understood in the context of our deterministic model, when all speakers are metropolitan and therefore experience the global properties of the system.

## Data accessibility

This article has no additional data.
